# Septicæmia

**Published:** 1890-08

**Authors:** Wm. Boll

**Affiliations:** Castroville


DANIEL'S

### TEXAS MEDICAL JOURNAL.

A Representative Organ of the Medical Profession, and an Exponent of Rational Medicine; devoted to the Organization, Advancement and Elevation of the Profession in Texas.

PUBLISHED MONTHLY.—SUBSCRIPTION $2.00 A YEAR.

Vol. VI. AUSTIN, AUGUST, 1890. No. 2.

### Original Articles.

CONTRIBUTED EXCLUSIVELY TO THIS JOURNAL.

*The Articles in this Department are accepted and published with the understanding that we are not responsible for, nor do we indorse the views and opinions of the writers by so doing.*

### SEPTICÆMIA.

BY DR. WM. BOLL, CASTROVILLE.

[Read at Quarterly Meeting West Texas District Medical Society, and voted to Daniel's Texas Medical Journal for Publication.]

WITHOUT entering into a detailed history of septicæmia, it is generally known that at the time of Hypocrates, it was distinguished as a disease originating from wounds, and characterized by a specific fever.

Injuries like lacerated wounds, compound fractures, etc., were known to be followed by inflammatory or traumatic fever, corresponding to the lesions more or less severe. As the etymology of septicæmia signifies, it means a certain condition of the blood in the system, which has been brought on by the absorption of putrid or decomposing substances. It is well known that the result of a lacerated wound will always be accompanied by decomposition of those parts that have been cut off from the proper supply of blood, and that before the process of granulation appears, some of this decomposed material might be absorbed and carried on into the general circulation.

That the lymphatic vessels play an important part in taking up those deleterious substances is doubtless, but it seems that they close soon, however, after the injury has been received, and when the inflammatory new formation sets in, there are no more open lymphatic vessels. Those were at least the views about thirty years ago.

But the absorption is not connected only to the lymphatics. It has also been proved, that capillaries and veins take an important part in the process of osmosis. Their walls would be permeated by liquids, and in this way it is very possible that putrid substances in a state of solution will enter into the general circulation. It is therefore current, that even after the lymphatic vessels close up, the system may be infected (if not even during the process of granulation). There must be other causes however, to develop septicæmia, and those causes seem to play a more important part than the above mentioned. It is the introduction of living organized bodies of a very minute size in the blood, that started the so called "germ theory." The existence of micrococcus a small body of a cell shape with a nucleus in the center, and of bacteria in the shape of a rode as minute organisms have been proved by microscopic researches for twenty years past. I am not able now to follow the latest investigations that have been developed through this new science, "bacteriology," which itself is yet in its childhood; but it is doubtless to me, that the etiology of septicæmia is in close connection with it. However, it has not been proved yet, that bacteria alone without putrid substances in solution would • infect the system. But it is more probable that the two causes are together, and that in fact, putrid substances are the most favorable condition for the development of the so-called microbes or protozoæ. After Kock's latest experiments with animals, it seems to be proved that after injections of putrid liquid, without the presence of bacteria, septicæmia has been developed. Those animals that died soon afterwards showed no signs of these organisms. But a certain number of them, that have been injected with only small quantities of putrid fluids, died later on, and in them bacteria seemed to appear in the course of the infection.

From the simple form of inflammatory or traumatic fever as Billroth describes it, to septicæmia is only a small step. Pyæmia, however, the absorption of pus as it means, in the circulation, is to be distinct from septicæmia, although the two diseases might co-exist and form the so-called pyo-septicæmia.

The symptoms of septicæmia will soon manifest themselves after an injury has been received or the performance of a surgical operation. They resemble very much those of an attack of remittent fever. The frequency of the pulse will rise, the tongue, lips and throat become dry, the skin feels hot, and the bodily temperature will soon commence to get higher.

The patient is apt to be apathetic, is inclined to sleep and don't like to be disturbed. He may refuse to take nourishment, but don't object to drink, when offered to him. The stomach becomes very irritable. Nausea and vomiting are therefore very frequent, and sometimes very obstinate. As soon as we examine the wound, we may find it generally in an unhealthy condition; a bad odor or other signs of putrefaction will be noticed. The skin surrounding the wound is œdematous, of yellow, bluish or even blackish color or of that bronzed tone, the forerunner of the so much threatened gangrene. The second day we find the patient with even a more frequent pulse, which already commences to lose its strength. The temperature gets even higher with only small remissions, that occur more generally in the morning. The bowels are sometimes constipated, but more frequently a diarrhœa sets in. The evacuations are highly offensive, and often very watery and thin. The abdomen might get tympanitic like in typhoid fever. The urine is very scanty, concentrated, even oppressed and often loaded with albumen and deposits. The respiration is generally rapid and superfical. The appearance of the wound and its surroundings might be changed but very little, or the signs of approaching gangrene will become more prominent.

The third and fourth day will not be much different; there might be a small fall of temperature, perspiration will be noticed, and the tongue might get a little moist. Bed sores are also apt to appear now. A slight teterus [?] is often covering the whole body, and especially marked in the conjunctivæ. Occasionally a cough sets in combined with other symptoms of bronchial catarrh. Generally on the fifth day the temperature reaches its highest point, sometimes even 106° and 107°, and is marked now by a sudden fall to the normal point or even below, a day or two afterwards. The pulse gets very high in frequency, and is hardly perceptible at the wrist. The agony of approaching death sets in, and the patient dies from collapse. This is the termination from an acute attack of septicæmia, that is brought on by injuries of large extension, as compound, comminuted fractures, involving larger joints, etc. But in milder cases, where recovery generally takes place, the fever may keep on for weeks yet, similar to cases of remittent fever. Reconvalescence may sometimes be soon established, while in other cases it will be protracted and follow a chronic course.

The diagnosis of septicæmia cannot be misrepresented, if we take in consideration, that it is only of traumatic origin and is to be differentiated from pyæmia. Septicæmia is generally initiated soon after contraction of the injury; pyæmia commences only after suppuration of the wound has set in. The former usually sets in without any chill, while pyæmia is always marked in the onset by a severe rigor, that is repeated like in intermittents. The two diseases are analogous to a case of congestive intermittents as pyæmia, and remittent fever with a tendency of typhoid condition as septicæmia. In pyæmia we always find metastatic abscesses in the various parts of the body, in the lungs, liver, kidney and brain. Serous cavities are frequently filled up with purulent deposits, and similar deposits may be found on the joints.

In septicæmia, on the contrary, the post mortem appearance gives a negative result, with the exception of condition of the blood. The prognosis is always serious, and in proportion to the extent of the injuries received, and other circumstances.

For the treatment of septicæmia prophylactic measures play the most important role. Since Lister commenced to establish a new method for treating wounds and guide surgical procedures, based on the germ theory, many cases of septicæmia have been prevented. Antisepsis and asepsis now are recognized and adopted as safe measures to prevent septic fever. And now capital operations are performed without being followed by septicæmia. Union by first intention is now the motto in wounds made by the operative knife, where two decades ago a process of suppuration would necessarily follow. Among the antiseptic remedies, carbolic acid has been used very extensively in solutions of varying strengths, and a dressing for wounds and a general disinfectant. Bichloride of mercury also seems to be a favorite with many. Salicylic and boracic acids are also used with good results. Iodoform, iodal, carbolized iodine, thymol, salol, aristol, etc., have their advocates, and the list of antiseptic remedies is constantly augmented by chemical discoveries.

If septicæmic is already developed, we should not hesitate to use antiseptics vigorously, even if it is thought that here action would be too late. It may be so in pyæmia, but I know of a case of puerperal septic infection where antiseptic injections in the uterine cavity ameliorated the symptoms soon and saved the patient.

Antipyretics may be used internally. Quinine in combination with mineral acids seems to have a beneficial effect in some cases. Stimulants also are required, but the utmost cleanliness carried out properly in combination with antiseptics, and especially the use of sterilized water exclusively in operations like laparotomies, etc., have shown the best results so far.

---

Dr. B. H. Detwiler, of Williamsburg, Pa., read before the Lycoming Medical Society, recently, a paper in which he detailed at length his experience with Campho-Phenique in practice. In wounds, in ulcers, in parturition, and, in fact, in all cases where a reliable germicide and antiseptic is needed, he had the best reults, and says now that he prefers it to all others as an adjunct to treatment in all cases requiring antiseptics.

